# Circular RNAs Represent a Novel Class of Human Cytomegalovirus Transcripts

**DOI:** 10.1128/spectrum.01106-22

**Published:** 2022-05-23

**Authors:** Shaomin Yang, Xiaolian Liu, Mei Wang, Di Cao, Dabbu Kumar Jaijyan, Nicole Enescu, Jian Liu, Songbin Wu, Sashuang Wang, Wuping Sun, Lizu Xiao, Alison Gu, Yaolan Li, Hong Zhou, Sanjay Tyagi, Jianguo Wu, Qiyi Tang, Hua Zhu

**Affiliations:** a Jinan University, Guangzhou, Guangdong, China; b Department of Microbiology and Molecular Genetics, New Jersey Medical School, Rutgers University, Newark, New Jersey, USA; c School of Biological Sciences and Biotechnology, Minnan Normal University, Zhangzhou, Fujian, China; d Department of Pain Medicine and Shenzhen Municipal Key Laboratory for Pain Medicine, Shenzhen Nanshan People's Hospital, The 6th Affiliated Hospital of Shenzhen University Health Science Center, Shenzhen, Guangdong, China; e Department of Microbiology, Howard University College of Medicine, Washington, DC, USA; f Public Health Research Institute, New Jersey Medical School, Newark, New Jersey, USA; g Department of Medicine, New Jersey Medical School, Rutgers University, Newark, New Jersey, USA; Wuhan Institute of Virology

**Keywords:** cytomegalovirus, HCMV, EBV, KSHV, circRNA, RNA splice junction

## Abstract

Human cytomegalovirus (HCMV) infects a large portion of the human population globally. Several HCMV-derived noncoding RNAs are involved in the regulation of viral gene expression and the virus life cycle. Here, we reported that circRNAs are a new class of HCMV transcripts. We bioinformatically predict 704 candidate circRNAs encoded by the TB40/E strain and 230 encoded by the HAN strain. We also systematically compare circRNA features, including the breakpoint sequence consensus, strand preference, length distribution, and exon numbers between host genome-encoded circRNAs and viral circRNAs, and showed that the unique characteristics of viral circRNAs are correlated with their genome types. Furthermore, we experimentally confirmed 324 back-splice junctions (BSJs) from three HCMV strains, Towne, TB40/E, and Toledo, and identified 4 representative HCMV circRNAs by RNase R treatment. Interestingly, we also showed that HCMV contains alternative back-splicing circRNAs. We developed a new amplified FISH method that allowed us to visualize circRNAs and quantify the number of circRNA molecules in the infected cells. The competitive endogenous RNA network analysis suggests that HCMV circRNAs play important roles in viral DNA synthesis via circRNA-miRNA-mRNA networks. Our findings highlight that circRNAs are an important component of the HCMV transcriptome that may contribute to viral replication and pathogenesis.

**IMPORTANCE** HCMV infects 40% to 100% of the human population globally and may be a life-threatening pathogen in immunocompromised individuals. CircRNA is a family of unique RNA that is the most newly found and remains unknown in many aspects. Our current studies computationally identified HCMV-encoded circRNAs and confirmed the existence of the HCMV circRNAs in the infected cells. We systematically compared the features between host and different viral circRNAs and found that the unique characteristics of circRNAs were correlated with their genome types. We also first reported that HCMV contained alternative back-splicing circRNAs. More importantly, we developed a new amplified FISH method which allowed us for the first time not only to visualize circRNAs but also to quantify the number of circRNA molecules in the infected cells. This work describes a novel component of HCMV transcriptome bringing a new understanding of HCMV biology and disease.

## INTRODUCTION

Human cytomegalovirus (HCMV) is a large double-stranded DNA virus that belongs to the beta-herpesvirus subfamily. HCMV infects humans at a rate of 40% to 60% in developed countries and 80% to 100% in developing countries (1, 2). During the primary infection, HCMV replicates in the epithelial cells of the initially infected organ and can spread to other organs through the blood (3). Primary HCMV infection is mostly asymptotic in immune-competitive people and causes nonspecific influenza-like symptoms in about 5% to 20% of people (3). However, in immunocompromised people, including newborns, organ transplant patients, and AIDS patients, the latent HCMV may reactivate causing congenital abnormalities and severe life-threatening complications (3). Many clinical HCMV strains have been isolated for studies in the laboratory, such as laboratory HCMV strains (Towne and AD169) and clinical strains (TB40/E, HAN, and Toledo). They display different levels of virulence, tissue tropisms, and pathogeneses (4). The major difference between laboratory and clinical strains is that the laboratory strains lose a 13 to 15 kb multigene segment, resulting in altered tropisms and reduced pathogenicity (5). The HCMV genome is approximately 230 kb in length and consists of unique long (UL) and unique short (US) segments, harboring approximately 225 open reading frames (ORFs) and terminal repetitive sequences (TRSs) (6). HCMV also encodes polyadenylated noncoding RNAs, such as RNA2.7, RNA1.2, RNA4.9, and RNA5.0 (7). In addition, numerous HCMV micro-RNAs have been identified and are involved in regulating the virus life cycle (8). For several years, newly noncoding RNAs encoded by HCMV have been sparsely reported.

Among the noncoding RNAs, circular RNAs (circRNAs) are a family of unique RNAs that are the most newly found and remain unknown in many aspects. CircRNAs have a covalently closed configuration that distinguishes them from all other RNAs. CircRNAs are formed by covalently bonded single-stranded RNA through back splicing and lack a cap at the 5′ end and 3′ end poly (A) (9). Unlike the formation of mRNA by polymerase II, tRNA or 5srRNA by polymerase III, and large rRNA by polymerase I, it is unknown what enzyme is involved in the formation of circRNAs. However, circRNAs have been identified in all kinds of cells and have been demonstrated to be associated with different diseases, implying that circRNAs must have important biological functions. The best-characterized functions of circRNAs include acting as microRNA (miRNA) sponges, playing the role of competitive endogenous RNAs (ceRNAs), and regulating the expression of parental genes in *cis* form. For example, human circRNA, circHIPK3, sponges miR-558 to regulate the growth of cancer cells (10) and plays an important role in diabetic neuropathic pain (11). In addition, a subset of circRNAs functions as a template for protein translation (12). Therefore, the biogenesis and biological functions of circRNAs have been topics of interest.

Besides the fact that circRNAs are found in many species across the eukaryotic kingdom, recent research has revealed a repertoire of circRNAs that are encoded by viruses. This may be explained by the fact that viruses are all obligate intracellular parasites. Despite the diversity in the abundance of viral circRNAs, it has been demonstrated that some viral circRNAs may play important roles in disease pathogenesis. Viral circRNAs have been computationally and experimentally detected for both DNA and RNA viruses. Interestingly, the most studied DNA viruses related to circRNAs are tumor viruses, including the Epstein Barr virus (EBV) (13–16), Kaposi Sarcoma herpesvirus (KSHV) (14, 17–19), and human papillomaviruses (HPVs) (20). We recently reported that circRNAs are an important component of the transcriptome encoded by RNA viruses of the betacoronavirus genus of Coronaviridae, severe acute respiratory syndrome coronavirus 2 (SARS-CoV-2), SARS-CoV, and Middle East respiratory syndrome (MERS-CoV) (21).

In this study, we unprecedently identified circRNAs derived from HCMV transcriptomes through bioinformatics and systematically compared their characteristics with those of the host and other DNA viruses. We further verified the bioinformatically predicted circRNAs using RT-PCR and RNase R sensitivity assays. In addition, we annotated the functions of HCMV circRNAs based on theories of RNA competition networks and the regulation of parental genes. This work has further expanded the transcriptome of HCMV and lays the foundation for understanding new mechanisms by which HCMV promotes infection, latency, and pathogenesis.

## RESULTS

### Computational identification of HCMV-derived back-splicing junction reads.

To look for potential HCMV-encoded circRNAs and compare their characteristics with other DNA viral circRNAs, we analyzed the deep RNA sequencing data sets that are publicly available from the NCBI GEO database for HCMV TB40/E strain-infected HFFs (primary fibroblasts), EC (endothelial cells), NPC (neural progenitors) derived from embryonic stem cells (22), and HCMV HAN-strain-infected HELF (human embryonic lung fibroblasts) cells exposed to RNase R-treatment to enrich circular RNAs by selectively degrading linear RNAs (Table 1). To increase the identification accuracy, biological replicates of the same condition were pooled. After running the BWA-MEM pipeline, 380,468,862, 447,601,571, 376,536,183, and 169,736,472 total reads were obtained from the TB40/E-strain-infected EC, NPCs, HFFs, and HAN-strain-infected HELF cells, respectively, with 53.06%, 24.11%, 52.95%, or 3.52% of the reads mapping to the HCMV genome, respectively (Fig. S1A to D and Fig. S1G to J). The genome coverage of the HAN strain with RNase R-treatment showed a sharp peak at 155,000 to 165,000 bp, suggesting that several HCMV RNAs transcribed from 155,000 to 165,000 bp are resistant to RNase R treatment (Fig. S1J).

**TABLE 1:** Information on the RNA-seq data set used in this study

Virus	Strain	Reference	Cell types	RNase R^*a*^	NCBI GEO ID	NCBI SRA ID	Layout	cDNA library	Bases (Gb)	Read length
HCMV	TB40/E	KF297339.1	EC	−	GSE73853	SRR2747466	Single	PolyA selected	17.7	100
HCMV	TB40/E	KF297339.1	EC	−	GSE73853	SRR2747467	Single	PolyA selected	20.8	100
HCMV	TB40/E	KF297339.1	NPCs	−	GSE73853	SRR2747469	Single	PolyA selected	23.3	100
HCMV	TB40/E	KF297339.1	NPCs	−	GSE73853	SRR2747470	Single	PolyA selected	21.9	100
HCMV	TB40/E	KF297339.1	HFF	−	GSE73853	SRR2747463	Single	PolyA selected	19.5	100
HCMV	TB40/E	KF297339.1	HFF	−	GSE73853	SRR2747464	Single	PolyA selected	18.5	100
HCMV	HAN	KJ426589.1	HELF	+	GSE138836	SRR10277187	Paired	Ribosomal RNA removed	25.5	150
KSHV	BCBL1	HQ404500	B cell	+	GSE117798	SRR7611495	Paired	Ribosomal RNA removed	12.9	150
KSHV	BCBL1	HQ404500	B cell	+	GSE117798	SRR7611496	Paired	Ribosomal RNA removed	11.6	150
EBV	Akata	KC207813.1	B cell	+	GSE116675	SRR7474066	Paired	Ribosomal RNA removed	11	100
EBV	Akata	KC207813.1	B cell	+	GSE116675	SRR7474067	Paired	Ribosomal RNA removed	17.3	100
EBV	Akata	KC207813.1	B cell	+	GSE116675	SRR7474068	Paired	Ribosomal RNA removed	18	100

a +, RNase R treatment; −, without RNase R treatment.

**FIG 1:**
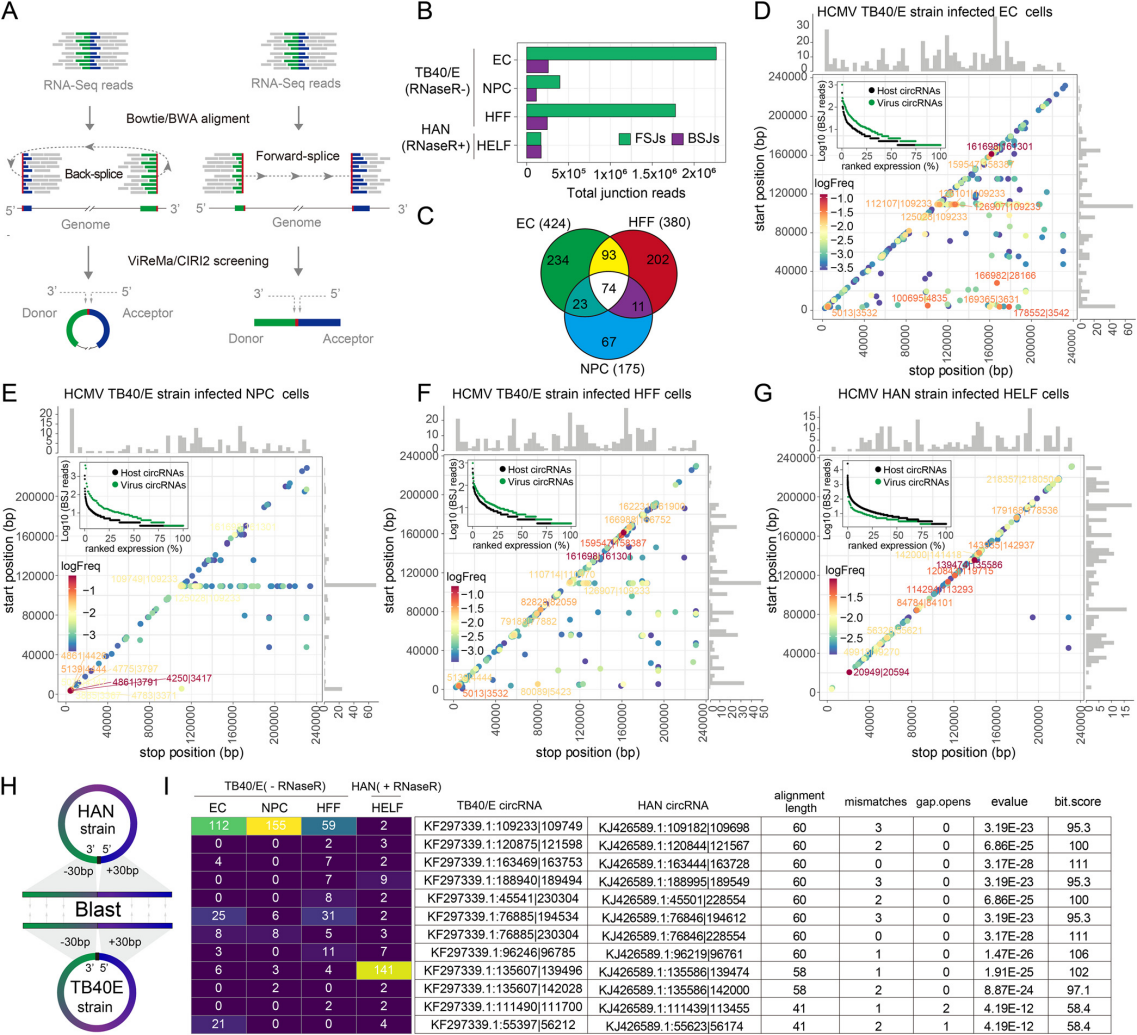
Predicted HCMV circRNAs and their abundances and landscapes. (A) Schematic diagram of CIRI2-based identification of circRNAs. Junction reads are aligned to the donor and acceptor sequences. If the genomic location of the donor is downstream of the acceptor, it is considered a back-splice junction (BSJ). The 5′ and 3′ breakpoints are then determined. (B) ViReMa-based identification of HCMV FSJs and BSJs from HCMV-infected cells. (C) Venn diagram presenting the number of unique or shared circRNAs in each paired group. (D to G) Frequency of circularization events in HCMV TB40/E-strain-infected EC (D), NPCs (E), HFF (F), and HAN-strain-infected-HELF cells (G). Counts of BSJ-spanning reads of circRNAs (starting from a coordinate on the *x*-axis and ending in a coordinate on the *y*-axis) are indicated by the colors. The numbers of circRNA start/stop positions are shown as histograms on the *x*-axis and *y*-axis. The ranked expression level of *de novo* identified circRNAs from HCMV and human genomes. The labels of the top 10 circRNAs are given according to their genome positions. The complete list of splice sites is provided in Data Set S2. (H) Schematic diagram of the alignment between the HAN and TB40/E strain circRNAs. (I) The alignment results of the top 12 conserved HCMV circRNAs from the infected cells.

Gatherer et al. (23) reported that HCMV encodes a large amount of RNA containing splicing junctions. ViReMa is a quick and sensitive tool for identifying viral RNA splice junctions, including forward-splice junctions (FSJs) and BSJs (Fig. 1A), from next-generation sequencing data (24). Therefore, we applied ViReMa to assess the abundance of HCMV-derived BSJs and FSJs. We obtained 100,424, 8,614, 27,530, or 43,187 splicing junction events from EC, NPC, or HFF cells infected with the TB40/E strain or HAN-strain-infected HELF cells (Fig. 1B). Our results showed that FSJs were more abundant than BSJs in TB40/E-strain-infected HFF, EC, and NPC cells, while they were significantly dissolved to the level of BSJs in HAN-strain-infected HELF cells that had undergone RNase R treatment.

Several circular RNA prediction algorithms have been developed to specifically identify these BSJs from RNA sequencing data, such as find_circ (25), CIRCexplorer2 (26), and CIRI2 (27). CIRI2 is an annotation-independent tool that is used for the high-precision identification of BSJs reads from BWA-MEM alignment output files (Fig. 1A) (27). Moreover, CIRI-full (28), a downstream analysis tool for CIRI2, is a powerful tool that is used for *de novo* reconstruction of full-length circRNAs.

The CIRI2 pipeline predicted 424, 380, and 175 HCMV circRNAs in the TB40/E strain-infected EC, HFF, and NPC, respectively, with 74 circRNAs being commonly expressed in the three infected cell types (Fig. 1C). We also identified 230 HCMV circRNAs from the HAN-infected HELF cells (Data Set S2). To examine the circRNA landscape, we mapped HCMV circRNAs from the 5′ and 3′ breakpoints of the BSJs to their respective genomic locations and estimated the back-splicing frequency by counting BSJ-spanning reads (Fig. 1D to G). In the data sets of the TB40/E strain without RNase R treatment, 62% of the shared 74 circRNAs (distance less than 4Kb) were local BSJs, while 32% of circRNAs, starting with the genome position of 109,233bp, were distant BSJs (Fig. 1D to F). The pattern of TB40/E circRNAs from HFF cells was more similar to that of undifferentiated EC cells than differentiated NPC cells. However, local BSJs were dominant in the circRNAs of the HAN strain in HELF cells that had undergone RNase R treatment (Fig. 1G). These results suggested that the expression patterns of HCMV circRNAs are different in different cell lines, and local BSJs are more tolerant to RNase R.

To further identify the conserved HCMV circRNAs generated from different types of cells infected with different strains of HCMV, we merged the HCMV circRNA BSJs from all infected cell lines and aligned the BSJ sequences between HAN and TB40/E strains using the BLAST aligner (Fig. 1H and Data Set S2). We found 12 circRNAs with over alignment lengths of 40 bp from 60 bp BSJ sequences that were conserved in both the HAN and TB40/E strains (Fig. 1I). Significantly, TB40/E KF297339.1:135607|139496 (HAN, KJ426589.1:135586|139474) was increased in HAN-strain-infected cells that had undergone RNase R treatment. These findings imply that conserved HCMV circRNAs may play an important part in HCMV infection.

### Conservative motif in the breakpoint of HCMV circRNAs.

**FIG 2:**
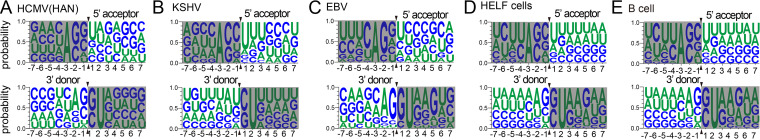
The breakpoint of HCMV circRNAs contains a conservative motif. The frequencies of 14 nt back-splice sequences of circRNAs identified in HCMV HAN strain (A), KSHV (B), EBV (C), HELF cells (D) and B cells (E).

It was reported that the splicing donor and acceptor sequences of human circRNAs have conservative splicing signatures: AG|GU (29). However, it is unknown whether there are any different splicing signatures for circRNAs that are transcribed from different genome types. To address this question, we compare the back-splice junction signatures of HCMV circRNAs with those of other DNA viruses and hosts. CIRI2 is dependent on BWA-MEM, which was originally designed for mapping DNA sequencing reads and can identify BSJs in circRNA AG|GU splicing signals in an unbiased manner (28). To ensure that the condition of these data sets was as consistent as possible, the data set with RNase R treatment and 100 to 150 nt length paired-end reads (Table 1) were selected for further analysis. We applied CIRI2 to further reanalyze KSHV, EBV, and their host cellular circRNAs (Fig. S1E and F, Fig. S1M and N, and Data Set S2) from the data sets of KSHV BCBL1 strain-infected B cell lymphoma cells that had undergone RNase R treatment (30) and EBV Akata strain-infected B cell lymphoma cells that had undergone RNase R treatment (13) (Table 1). We obtained 14 nucleotide sequences flanking the BSJ donor and acceptor sites. WebLogo analyses showed that HCMV circRNAs have the canonical “AGN|UNN” signal at the acceptor site and a noncanonical “NAG|GUN” sequence at the donor site. The A (−3) base and U (+2) base located in the 5′ and 3′ introns of HCMV circRNAs are extremely conserved (Fig. 2A), which is similar to what occurs in humans, KSHV, and EBV encoded circRNAs (Fig. 2B to E). Our results indicate that the back-splice junction signatures of HCMV circRNAs contain a conservative motif similar to that found in human cell lines, KSHV, and EBV.

### Systematic comparison of the characteristics of HCMV circRNAs with those of host and other DNA viral circRNAs.

**FIG 3:**
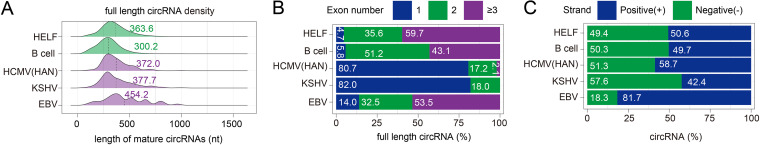
Characterization of HCMV circRNAs. (A) Length distribution of circRNAs in the host, HCMV HAN strain, KSHV, and EBV. The average lengths are indicated by dashed lines. (B) Statistics of the exon number of the host, HCMV HAN strain, KSHV, and EBV circRNAs. (C) Strand preferences of the circRNAs in the host, HCMV HAN strain, KSHV, and EBV. Number of full-length circRNAs used in the analysis: HELF cells, 19,850; B cells, 9705; HCMV, 145; KSHV, 283; and EBV, 43.

Next, we scrutinized the following characteristics of the HCMV circRNAs: length, exons contained, and strand preferences. It has been reported that the lengths of circRNAs of humans, macaques, mice, rats, rabbits, and chickens are highly conserved, with the majority having conserved lengths ranging from 250 to 500 nt (28). To further characterize HCMV, KSHV, EBV, and their host cellular circRNAs, we performed *de novo* reconstruction of full-length HCMV circRNAs using CIRI-full (28). We found 145, 283, and 64 full-length circRNAs in HCMV (Fig. S2), KSHV, and EBV, respectively, as well as additional partially assembled viral circRNAs (Data Set S2). The reconstruction of host circRNAs obtained 19,850 full-length circRNAs from HELF cells and 9705 full-length circRNAs from B lymphoma cells as well as additional partially assembled human circRNAs. The lengths of HCMV circRNAs were similar to those of other DNA viral circRNAs, and the average lengths of circRNAs of HCMV, KSHV, and EBV were 372.0, 377.7, and 454.2 nt, respectively, longer than those of the host (Fig. 3A).

To determine whether one or more exons were contained in the circRNAs of HCMV, KSHV, EBV, and host cells, we performed a statistical analysis of the exon numbers of the reconstructed full-length circRNAs. We found that 80% of HCMV and KSHV circRNAs tended to include a single exon, whereas over 86.0% of EBV and host circRNAs contained multiple circular exons (Fig. 3B). It may be interesting to investigate whether circRNAs express any proteins due to the inclusion of the exon.

In addition, we set out to examine whether the circRNAs had a strand preference. To that end, we performed a statistical analysis of the percentage of reconstructed full-length circRNAs. We found that HCMV circRNAs tended to be derived from positive-strand DNA as opposed to human circRNAs (Fig. 3C). Although both EBV and KSHV establish latency in B lymphoma cells (13, 31), the circRNA strand preference of these two tumor viruses differed. EBV circRNAs were mostly produced from positive-strand DNA, while KSHV tended to be from negative-stranded DNA (Fig. 3C). Therefore, viral circRNAs have a genomic strand preference.

### Experimental confirmation of the HCMV-encoded circRNAs.

**FIG 4:**
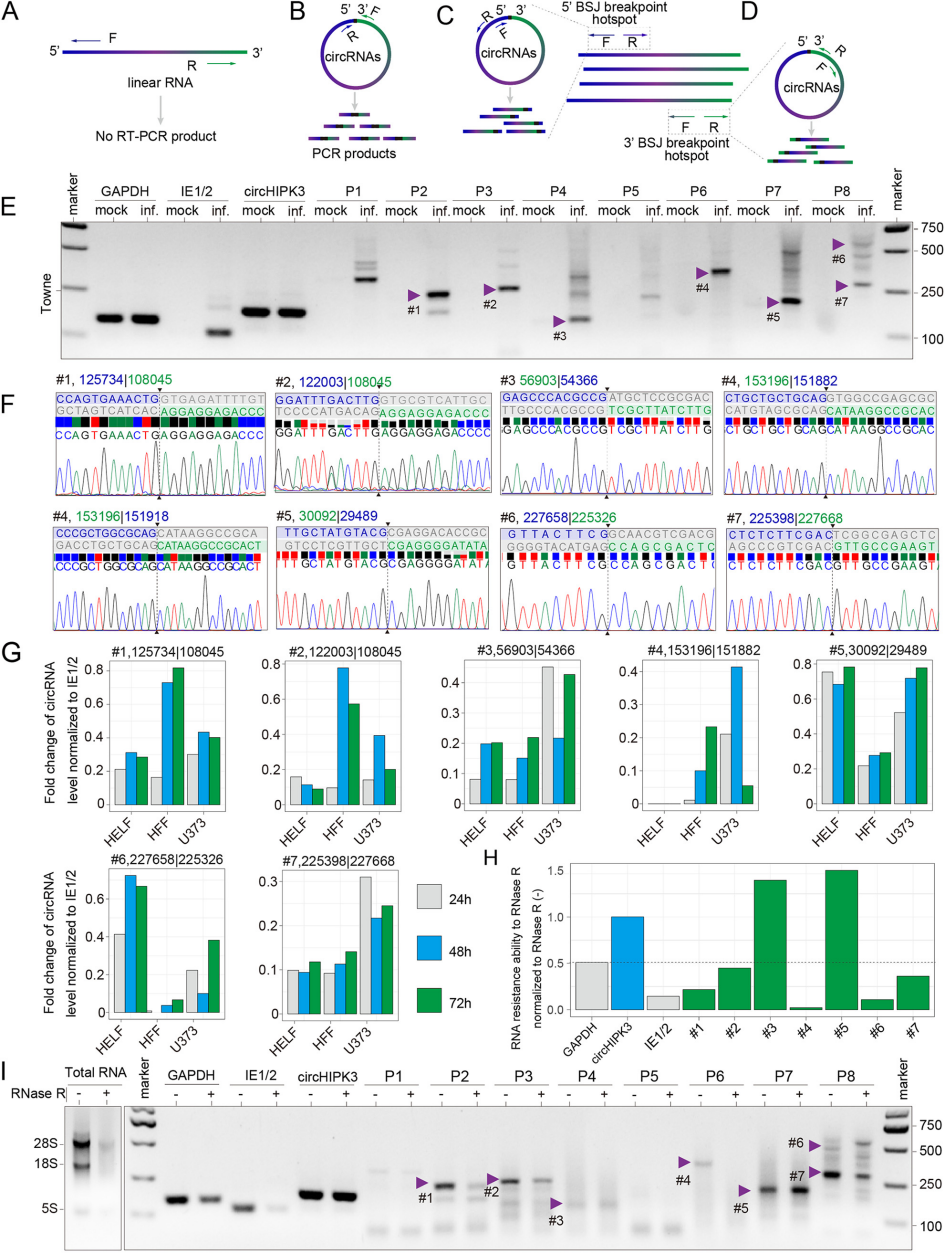
Experimental identification of HCMV circRNAs. (A) Illustration of how inverse RT-PCR with divergent primers selectively amplifies different regions of circRNAs but not linear RNAs. (B to D) Schematic diagram of how divergent primers were designed to amplify predicted BSJs (B) and confirmed full-length circRNA sequences (C and D). (E) Inverse RT-PCR result of the HCMV laboratory strain Towne with primer sets shown in (B). Bands indicated by arrows were sequenced. Representative Sanger sequencing results are shown in (F). BSJ breakpoints are indicated by dashed lines. Donor (green), acceptor (blue) sequences, and downstream/upstream sequences (gray) flanking the junction were aligned with the BSJ sequence. (H and I) Relative resistance ability to RNase R of linear RNA and circRNAs (G). The relative expression of HCMV circRNAs in HELF, HFF and U373 cells at different times of infection. The values were normalized to IE1/2. The value was normalized to the corresponding sample without RNase R treatment. (H). Bands are shown in (I), where the left panel shows an agarose gel analysis of total RNA with and without RNase R treatment, and the right panel shows the products of RT-PCR on RNA treated with and without RNase R. Human linear RNA, GAPDH, and HCMV linear RNA, IE1/2 (immediate early 1/2 gene, UL123) (35 cycles with convergent primers), Human circRNA, circHIPK3, and HCMV circRNAs (35 cycles with divergent primers).

To experimentally demonstrate the production of HCMV-encoded circRNAs, we extracted total RNA from HFF cells that were either infected or mock-infected with the HCMV clinical strains TB40/E or Toledo or a laboratory strain (Towne) at 72 hpi. Divergent primers were designed to amplify the targeted BSJs of the most abundant HCMV-encoded circRNAs (Fig. 4A and B) (Table 1). Additional two divergent primer sets were used to obtain the full length of circRNAs (Fig. 4C and D). To determine whether the inverse PCR products were from BSJs rather than nonspecific PCR products, we gel-purified candidate BSJ amplicons based on the molecular weight (Fig. 4E and Fig. S3A), subcloned and Sanger-sequenced at least 8 colonies for each candidate. We identified 7 BSJs from 8 primer sets (Table 1), and 6 BSJs were shared by the HCMV TB40/E, Toledo, and Towne strains (Fig. 4E, Fig. S3A, and violet arrowheads in Fig. 4F). The data prove that the three strains of HCMV generated circRNA in the infected cells. Furthermore, we used this pipeline to identify 324 HCMV BSJs of the Towne strain from 426 clones by systematically scanning most bioinformatically predicted circRNAs (Data Set S3). Next, we performed a systematic overview of over 400 articles reporting the functions of 172 HCMV ORFs and annotated 704 and 230 bioinformatically predicted HCMV circRNAs from the TB40/E and HAN strains and 324 experimentally identified BSJs from the Towne strain back to the HCMV genome reference (Data Set S4). Interestingly, the most abundant BSJs were found in the HCMV genome between 155,000 and 165,000 bp, which correlated with the sharp peak in the genome coverage of the HAN strain RNA-Seq data (Fig. S1J). Next, we detected the expression of circRNAs at different infection time points and in different cell lines (Fig. 4G and Fig. S3B and C). HCMV BSJs expression was detected in different cell lines, but HFF and U373 cells with the HCMV infection produced more viral BSJs than HELF cells and were more inclined to be in the late stage of infection.

CircRNAs have a covalently closed configuration and, hence, are more resistant to exoribonuclease RNase R than linear RNAs (9). To evaluate the tolerance of HCMV circRNAs to RNase R digestion, we selected 12 primer sets corresponding to 11 BSJs from 324 BSJs to perform a small sample RNase R sensitivity test. Agarose gel electrophoresis of total RNA extracted from Towne-infected HFF cells with or without RNase R treatment showed that most ribosomal RNAs were degraded after 20 min of RNase R treatment (Fig. 4I). While the linear viral RNAs derived from immediate-early (IE1/2), early (UL105), and late (UL111A) genes were completely degraded by RNase R, the local BSJ number 3 (FJ616285.1:56903|54366), BSJ number 5 (FJ616285.1:30092|29489, Fig. 4H and I) and the distance BSJ number 10 (FJ616285.1:227211|75707), BSJ number 11 (FJ616285.1:227213|44709, Fig. S3D to I) remained. However, bands corresponding to BSJ numbers 1 to 4 and BSJ numbers 6 to 9 were more susceptible. These results suggest that about 70% of BSJ-containing RNAs were not circularized. To further verify HCMV-encoded circRNAs, the full-length of circRNA number 5 with 640 nt was obtained, which BSJ number 5 signal could be detected by three different primer sets (Fig. S3J and Table 2). In conclusion, by using RT-PCR and the RNase R sensitivity assay, we confirmed that HCMV-infected cells contain virus-specific circRNAs.

**TABLE 2:** Primers and probes used in this study and full-length sequences of circRNA J616285.1:30092|29489

Primer set name	Primer name	Sequence (5′ to 3′)
Divergent primer		
circHIPK3	circHIPK3-F circHIPK3-R	TTCAACATATCTACAATCTCGGT ACCATTCACATAGGTCCGT
126350 | 108139	P1-F P1-R	AGAAACGCCGGTCGGTAAAA TCTTGGAAGCCGATGCAACA
125634 | 108139	P2-F P2-R	CTGACGTGCATGATTTGCCG TCTTGGAAGCCGATGCAACA
121883 | 108133	P3-F P3-R	CAGTCTTGCGGTTCCGTCTC AAGCCGATGCAACAACGGTA
56901 | 54433	P4-F P4-R	GCAATGACTGCGTACCGTTT GGCTCTTTGCTCGAAGGTGA
108485 | 108137	P5-F P5-R	ATAGCGCGTAGACGGACATC TTGGAAGCCGATGCAACAAC
153076 | 152061	P6-F P6-R	CCGACATCGTGGACAAATGC GTACGCCGATAACGACGACT
30026 | 29573	P7-F P7-R	CCTCAGCAGACGAGAGGATG TTCGTGGGTCGCTTCGTGAA
227402 | 225559	P8-F P8-R	CAGTTGCGAACGTCACCGGA GGTTCGCTAATCGCACGGAA
138288|134886	P9F	GCGAGAGGAAGTCGATCTGG
	P9R	TGTTGACGTCGTCCAGTGAG
108488|108179	P10F	GAGGAGACGACTGTCGGTAG
	P10R	AAGGTGCGGGAGACTAGGTC
227109|75825	P11F	AATGCGGTCCACCATCTTCA
	P11R	GTAACTTCAGTGGGCCCGTC
227109|45042	P12F	CGTGACTTGCTGGATCTCGT
	P12R	GTAACTTCAGTGGGCCCGTC
Convergent primers		
GAPDH	GAPDH-F GAPDH-R	GCACCGTCAAGGCTGAGAAC TGGTGAAGACGCCAGTGGA
IE1/2	IE1/2-F IE1/2-R	CCAAGAGAAAGATGGACCCTG AACATAGTCTGCAGGAACGTC
UL105	105F	GTGCCGGTATCTCAACGGAT
	105R	CGCGTACAGACTGGTGTGAT
UL111A	UL111AF	GTCTCTTCCTCTCTGGTCCTG
	UL111AR	CTTTCTCGAGTGCAGATAC
Probes for ampFISH		
DP-positive	GTTACAGACGACTCCCACAGTCCCGAGGGGATATAAATCACCGGACT	
AP-positive	GAGGGCTATGTTTTTTGCTATGTACGGGACTGTGGGAGTCGTCTGTAACTACTTCATGTTACAGACGACTCCCAC	
DP-negative	GTTACAGACGACTCCCACAGTCC CGTACATAGCAAAAAACATA GGACT	
AP-negative	CTCGACGGTGATTTATATCCCCTCGGGACTGTGGGAGTCGTCTGTAACTACTTCATGTTACAGACGACTCCCAC	
DP-linear 29489	GTTACAGACGACTCCCACAGTCC-CGAGGACACCGCCGTCTACT-GGACT	
AP-linear-29489	GAGGGCTATGTTTTTTGCTATGTACG -GGACTGTGGGAGTCGTCTGTAACTACTTCATGTTACAGACGACTCCCAC	
circHIPK3-ve DP	GTTACAGACGACTCCCACAGTCC GTATGGCCTCACAAGTCTTGGTCTGGACT	
circHIPK3-ve AP	ATATCTACAATCTCGGTACTACAG GGACTGTGGGAGTCGTCTGTAACTACTTCATGTTACAGACGACTCCCAC	
circHIPK3+ve DP	GTTACAGACGACTCCCACAGTCC CTGTAGTACCGAGATTGTAGATAT GGACT	
circHIPK3+ve AP	TAGACCAAGACTTGTGAGGCCATAC GGACTGTGGGAGTCGTCTGTAACTACTTCATGTTACAGACGACTCCCAC	
Full-length sequences of circRNA J616285.1:30092|29489		
circRNAnumber 5	CCTCGCGTACATAGCAAAAAACATAGCCCTCGTCCGAGATGAGGCACACAGCGGTCTTCTTCTGCTGATCCGGCGACAACACGCCCTCGTTCACGAAGCGACCCACGAAGGCCAGGCGCGTCTGGCAACACAGGTAGTGACTCCAAGCCTTCACGTCCTCCGGTTTGAAGTCCTCGTCCGTCTCGATCTCCTGCAGCACTAGGTTCCAGCCCGGCGGCCAGACCACGGGCAACACCTGGCCTGCGTTGATGCGCACGTAAGCTTCCAGACAGCCCAGGCCGAACTCGGCCGTGAGCGCCAGGCTAGCCAGATCGCTCATGTGACGCGCCGAGTCAGTGGGCGAGCCCGGGGGCCCGTCGCACACCACGCTCCGTCTTCTTGTCCTCACCGCGGCCAGCGTGGCGAGGACACTTTCCGCGCCCGAGGCTGTATCTTCGGTTTGCCCGCCGGAGCCGGCCCTCACTATATAACGTCCCGCCCGGGTCTCCTCCATGTATGCAGGTAAGCAACTGAGCCGAACGCACCTCAGCAGACGAGAGGATGTCGTCGCGGCGTCGCAGCTCGTCACGTCGCTCTGGCGAACCCTCGACGGTGATTTATATCC	

### Further verification of HCMV-encoded circRNA by amplified FISH (ampFISH).

**FIG 5:**
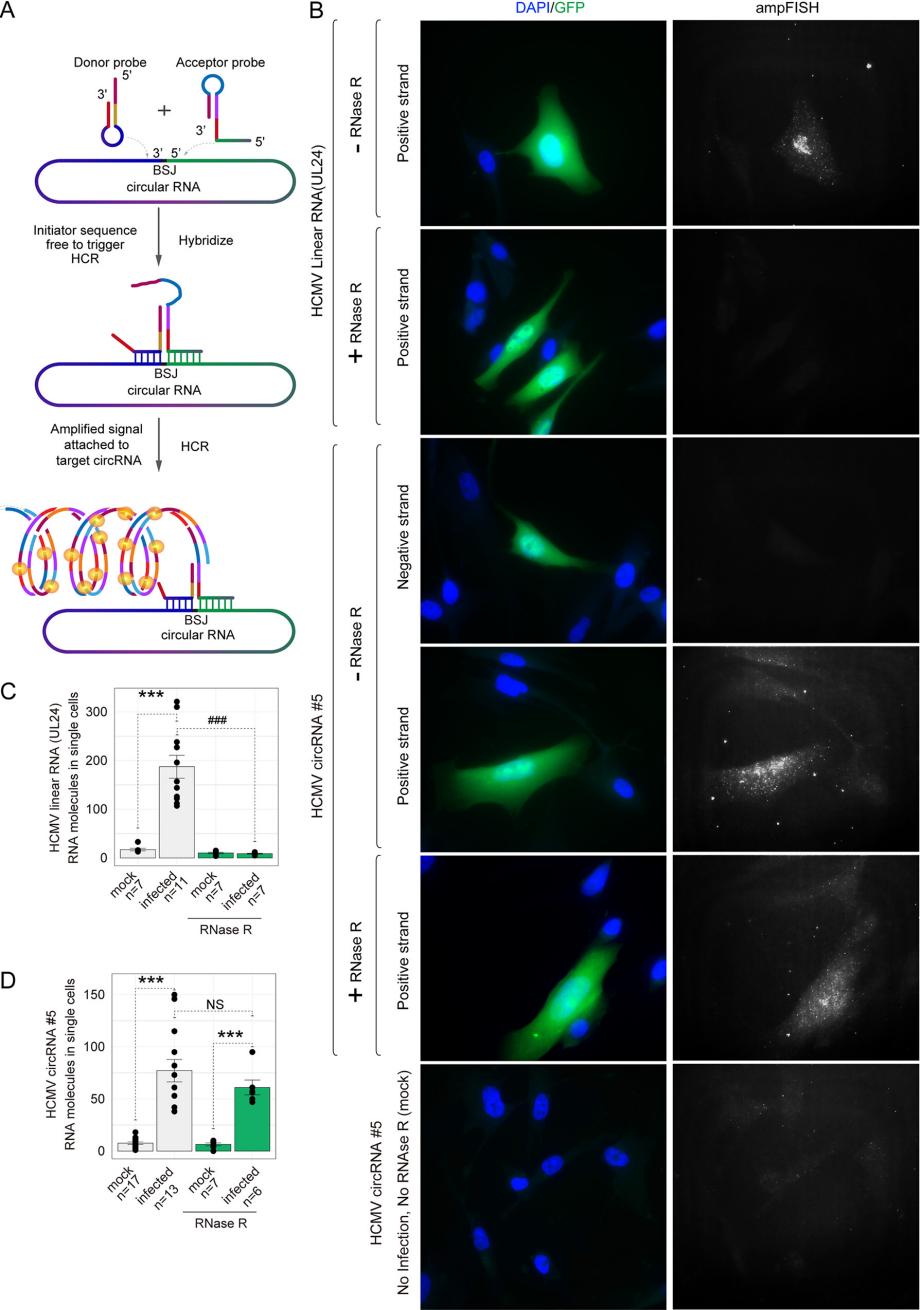
Detection of an HCMV circRNA *in situ* using ampFISH. (A) A schematic illustrating ampFISH. When two hairpin-shaped probes bind at adjacent locations on a target RNA, conformational reorganization occurs in one of them, which initiates a hybridization chain reaction (HCR) that deposits fluorescence at the target RNA molecule. The target sequences of the two probes are present next to each other in the circular RNA but not in the linear RNAs. (B) Representative HCMV-infected MRC5 cells were imaged after ampFISH either against linear RNA UL24 or circular RNA number 5 (FJ616285.1:30092|29489). Cells harboring HCMV are GFP-positive. ampFISH images are maximal intensity merges of z-stacks acquired in the Cy5 channel and presented under a common contrast level. (C and D) The number of ampFISH spots corresponding to single mRNA molecules counted in single cells for the indicated categories of cells. The evidence of the accuracy of spot counting is presented in Fig. S4. Two-tailed unpaired *t* test. *, *P* < 0.05; **, *P* < 0.01; ***, *P* < 0.001 mock versus infected group; ###, *P* < 0.001 without RNase R treatment versus with RNase R treatment group. Data are presented as mean ± S.E.M. The number of cells analyzed ranged from 6 to 16.

AmpFISH is a recently developed method with high fidelity for the detection of RNA expression and quantification in cells (Fig. 5A). The ampFISH signal is detectable only when the two probes are hybridized to adjacent positions on a target mRNA (32). To confirm the RT-PCR results, we performed ampFISH against HCMV circRNAnumber 5 and designed two pairs of probes to specifically target HCMV linear RNA UL24 and HCMV circRNAnumber 5 (Table 2). Imaging with these probes showed that HCMV UL24 linear RNA is localized in both the nucleus and cytoplasm of infected cells with a higher density in the nucleus, while uninfected cells showed little or no ampFISH signal (Fig. 5B). In contrast, probes targeting the sense back-splice junction sequence of HCMV circRNA number 5 gave clear signals that were spread throughout the infected cells. We also prepared a pair of probes targeting the antisense back-splice junction sequence of HCMV circRNA number 5, which showed no ampFISH signal (Fig. 5B), indicating that HCMV circRNAnumber 5 is generated from positive-strand DNA. Uninfected cells yielded no signals in any of the three sets of probes, demonstrating the specificity of signals. To further confirm that the ampFISH signals detected originated from circRNAs, we treated the fixed and permeabilized cells with RNase R and counted the number of spots in single cells (additional details presented in Fig. S4A) (32). Accordingly, the signals from probes targeting the UL24 linear RNA were lost, but the signals from probes targeting circRNA number 5 positive-strand remained, further confirming that the target RNA is circular (Fig. 5C and D).

To demonstrate that ampFISH signals are not artifacts of HCMV infection and that ampFISH accurately reports the presence of circular RNAs in general, we targeted the donor and acceptor sequences of a well-known type of endogenous circular RNA, circHIPK3 (Table 2). As a control, we also imaged the linear version of this RNA using a set of 48 smFISH probes (Fig. S4B). We found that signal amplification occurred only when the probes targeted the sense strand of the circHIPK3 back-splice junction sequence (Fig. S4B), which corresponds with the findings of Wen et al. (33). We then counted the number of spots that correlated with single RNA molecules in single cells. Interestingly, the results of both RT-PCR and ampFISH showed that the expression of circHIPK3 is upregulated by HCMV infection (Fig. S4C and D) and that RNAs R treatment leads to loss of linear RNA but not circular HIPK3 RNA. These results confirm the specificity of the ampFISH approach for detecting circular RNAs. This is the first time that circular RNAs have been visualized and quantified *in situ*.

### High-frequency reverse complementary and homology sequence in the breakpoint of HCMV circRNAs.

**FIG 6:**
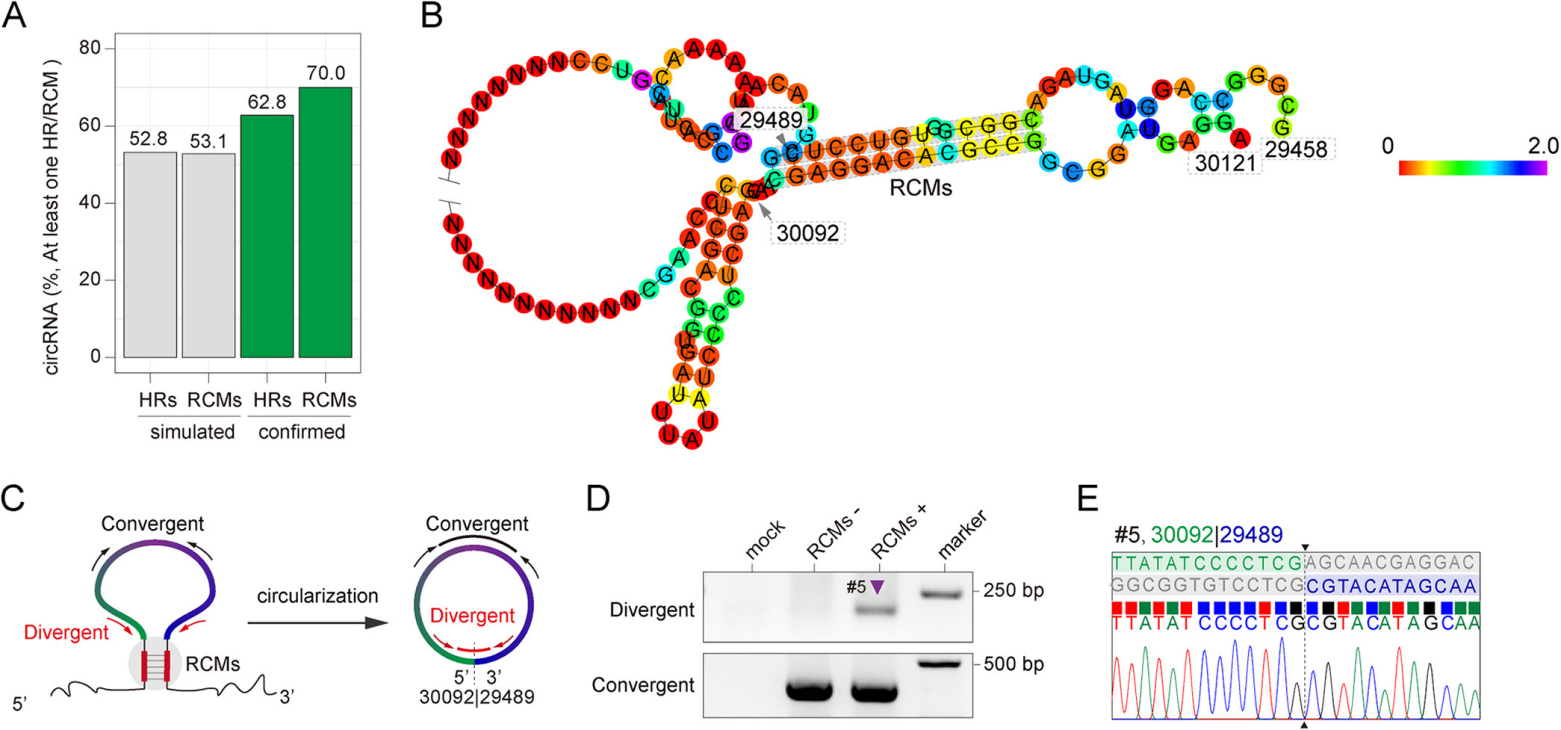
Homology and reverse complementarity sequence statistics. (A) The percentage of homology and reverse complementarity sequences in the simulated circRNAs and experimentally confirmed HCMV circRNAs. (B) RNA structure prediction for HCMV genomic region FJ616285.1:29458|30121. (C) Illustration of divergent primers selectively amplify circular form circRNA number 5, while convergent primers amplify both linear and circular form circRNA number 5. (D) Inverse RT-PCR result of 293T cells transfected with the regions FJ616285.1:29489|30092 and FJ616285.1:29458|30121 linear form expression vector. (E) Representative Sanger sequencing results were shown in (D). BSJ breakpoints are indicated by dashed lines. Donor (green), acceptor (blue) sequences, and downstream/upstream sequences (gray) flanking the junction were aligned to the BSJ sequence.

It has been reported that reverse complementary sequences (RCMs) between introns bracketing an exon are the cis-acting elements that promote the circularization of circRNAs (34). To determine whether any homology sequences (HRs) or RCMs flank the 5′ and 3′ breakpoints of HCMV circRNAs, we randomly simulated 324 HCMV circRNAs based on the HCMV genome and known average length of HCMV circRNAs (Data Set S3). The sequence analysis showed that these simulated circRNAs contained 52.8% HRs and 53.1% RCMs (Fig. 6A and Data Set S3). However, it was experimentally confirmed that the HCMV BSJs had 62.8% HRs and 70.0% RCMs. These data indicate that the sequence flanking the 5′ and 3′ breakpoints of HCMV circRNAs tends toward homology and reverse complementarity.

To examine how the HCMV circRNAs form, we analyzed the circularization of circRNA number 5, which contains 13 nt RCMs flanking the breakpoints (Fig. 6B). We transfected 293T cells with a plasmid expressing the linear form of circRNA number 5 that either contained the RCMs (FJ616285.1:29489|30092) or did not contain the RCMs, which replaced RCMs with random sequences. Convergent primers were designed to amplify both linear RNA and circular RNA, whereas divergent primers targeted specially BSJ number 5 (Fig. 6C). The results from the inverse RT-PCR showed that RCMs promoted the circularization of circRNA number 5 by back splicing (Fig. 6D). Furthermore, the Sanger sequencing results demonstrated that the RCMs could successfully and accurately create BSJ number 5 (Fig. 6E). However, the control that replaced RCMs with random sequence showed no amplify the signal by divergent primers. These data indicated that RCMs around the breakpoint of circRNAs serve as cis-acting elements, contributing to the biogenesis of HCMV circRNAs.

### Identification of alternative back-splicing of HCMV circRNAs.

**FIG 7:**
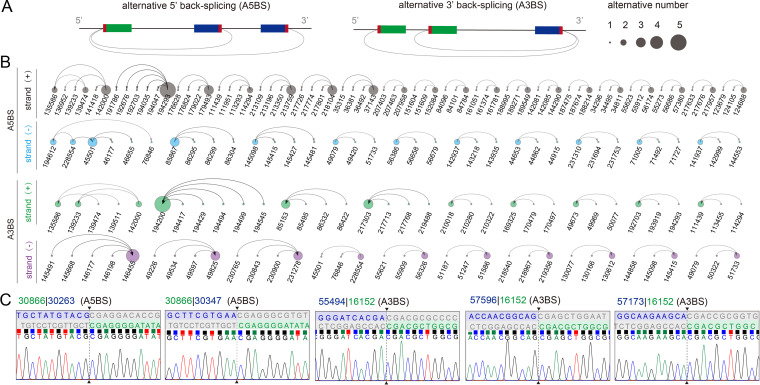
ABS events of circRNAs are prevalent in HCMV. (A) Schematic diagrams of two types of ABS events. (B) Alternative back-splice analysis of HCMV circRNAs. (C) Alternative back-splice circRNAs identified by RT-PCR. Breakpoints are indicated by dashed lines. Donor (green), acceptor (blue) sequences, and downstream/upstream sequences (gray) flanking the junction were aligned to the BSJ sequence.

Generally, a gene locus can produce multiple circRNAs that share the same breakpoints. This is called alternative back-splicing (ABS) (26, 35). ABS is divided into two types, according to the shared breakpoints, alternative 5′ back-splicing (A5BS) and alternative 3′ back-splicing (A3BS), as shown in the Fig. 7A. To evaluate the ABS events in HCMV circRNAs, we scanned all bioinformatically predicted HCMV HAN circRNAs and found 31 A5BS events and 20 A3BS events (Fig. 7B). Moreover, 13 A5BS events and 22 A3BS events were found from experimentally identified HCMV BSJs (Table S2 and Fig. 7C). These results indicated that HCMV encodes alternative back-splicing circRNAs.

### Functional analysis of HCMV circRNAs in a competitive endogenous RNA coregulatory network.

**FIG 8:**
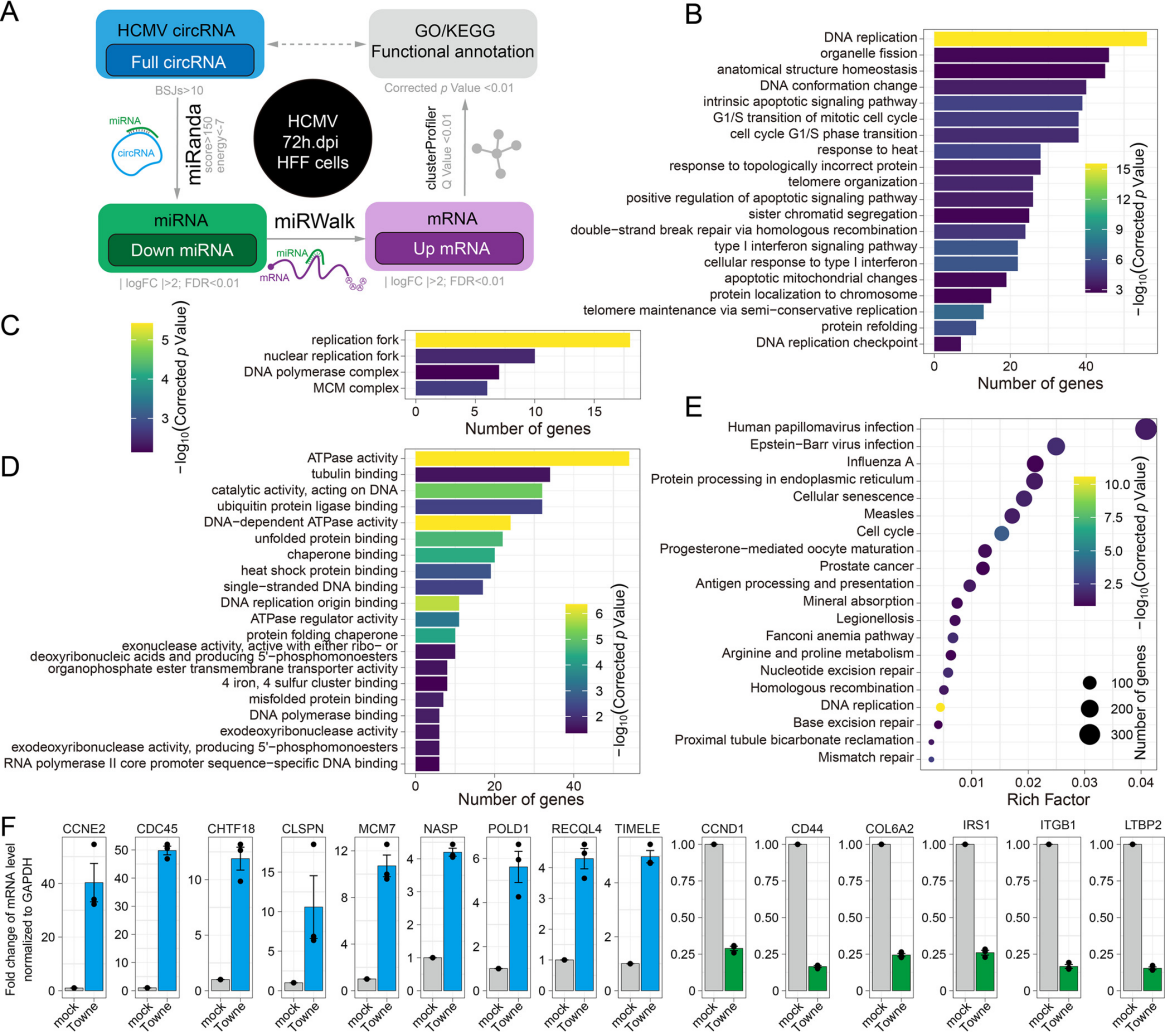
Potential HCMV circRNAs function in the competitive endogenous RNA coregulatory network. (A) Schematic of potential HCMV circRNA competitive endogenous RNA coregulatory network analysis method. (B to D) Comparison of Gene Ontology (GO) enrichment of HCMV-circRNA-associated genes that are upregulated upon HCMV infection. The figure shows the top 20 significantly enriched GO terms, including biological processes (B), cellular components (C), and molecular functions (D). (E) KEGG pathways of HCMV-circRNA-associated genes that are upregulated upon HCMV infection. (F) The mRNA expression levels of HCMV-circRNA-associated genes that are upregulated upon HCMV infection (*NASP*, *POLD1*, *CLSPN*, *MCM7*, *RECQL4*, *CHTF8*, *CDC45*, *TIMELE*, and *CCNE2*). Genes that are downregulated upon HCMV infection (*CCND1*, *CD44*, *COL6A2*, *IRS1*, *ITGB1*, and *LTB2*) were used as negative controls using quantitative real-time PCR.

Most RNA transcripts, including circRNAs, mRNAs, and long noncoding RNAs (lncRNAs), contain miRNA response elements (MREs) that compete during miRNA binding to regulate each other’s expression levels. This is known as competitive endogenous RNA (ceRNA) (36). Many reported circRNAs function as miRNA sponges and are also involved in the ceRNA regulatory network (37). To investigate whether gene expression is regulated by HCMV circRNAs via the miRNA sponging pathway, we performed a circRNA-miRNA-mRNA network analysis (Fig. 8A). First, we predicted that 145 highly expressed full-length HCMV circRNAs would interact with 1518 human miRNAs, as shown in Data Set S5, as HCMV-circRNAs-miRNAs. Second, we screened the mRNA targets of HCMV-circRNAs-miRNAs and found 19,073 HCMV-circRNAs-miRNAs-mRNAs. Third, we screened 341 differentially expressed HCMV-circRNAs-miRNAs as well as 2113 differentially expressed HCMV-circRNAs-miRNAs-mRNAs following HCMV infection based on published data sets (25). Finally, 991 upregulated HCMV-circRNAs-miRNAs-mRNAs that interact with downregulated HCMV-circRNAs-miRNAs were selected for gene ontology (GO) and KEGG pathway analyses.

The GO Biological Processes analysis revealed that HCMV circRNAs that upregulate gene expression following HCMV infection were mainly associated with “DNA replication” (Fig. 8B). The GO cellular component analysis also revealed the enrichment of the “replication fork” (Fig. 8C). The molecular function analysis identified enrichments in “ATPase activity” and “DNA-dependent ATPase activity” (Fig. 8D). In addition, the KEGG pathways analysis showed that upregulated genes associated with HCMV circRNAs are involved in “DNA replication” and the “cell cycle” pathway (Fig. 8E). Next, we validated 7 upregulated genes associated with HCMV circRNAs (*NASP*, *POLD1*, *CLSPN*, *MCM7*, *RECQL4*, *CHTF8*, *CDC45*, *TIMELE*, and *CCNE2*), which were associated with the most significant GO term and KEGG pathway (Fig. 8F and Fig. S5). These results suggest that HCMV circRNAs may act as cellular miRNA sponges to promote viral DNA synthesis.

## DISCUSSION

HCMV infection is the leading infectious cause of congenital disorders in newborns. Congenital HCMV infection causes permanent neurological and neurocognitive disabilities and results in significant health problems worldwide (1, 38–41). In addition, HCMV remains one of the most significant opportunistic pathogens encountered in patients with AIDS and organ transplantation recipients and is usually associated with gastroenteritis, pneumonia, retinitis, and transplantation failure (39, 42–44). No vaccine is available. The drugs currently used against HCMV are the synthetic acyclic analog of 2′-deoxyguanosine and its derivatives. Drug resistance frequently occurs, and no currently available drugs can be used for fetal exposure (45, 46). A novel and specific interference with viral replication is needed to treat HCMV infection, especially congenital HCMV infection. A full understanding of the HCMV transcriptome, which is currently lacking, is vital for developing new therapeutic strategies against HCMV.

Although varied noncoding RNAs have been identified from HCMV-infected cells, it is still unknown whether the HCMV genome encodes circRNA, a covalently circularized type of single-stranded noncoding RNA with versatile biological functions. CircRNAs have been identified from different eukaryotes. CircRNA profiling shows that many human circRNAs were involved in the pathogenesis of HCMV infection (47, 48). Recently, virus-encoded circRNAs were identified from infected cells. These viruses include KSHV, EBV, HPV, HBV, and coronaviruses (13, 17, 49–51). In the present study, we employed an unbiased algorithm, CIRI2, to analyze existing data sets to identify circRNAs not only for HCMV but also for EBV and KSHV. We unprecedently revealed that HCMV also expresses circRNAs in different cells and identified more circRNAs for EBV and KSHV than found by previously published studies (Data Set S2). CircRNA BSJ analyses of RNA-seq data sets that had undergone RNase R-treatment, including HAN-strain-infected HELF cells (Fig. 1G), KSHV-infected B cell lymphoma cells (Fig. S1M), and EBV-infected B cell lymphoma cells (Fig. S1N), showed mostly local BSJs, whereas circRNA BSJs from RNA-seq data sets that had not undergone RNase R-treatment (TB40/E strain-infected EC [Fig. 1D], NPCs [Fig. 1E], and HFFs [Fig. 1F] cells) had more distant BSJs. These results imply that local BSJs of DNA viral circRNAs are more resistant to RNase R than distant BSJ events.

We computationally analyzed the available RNA-seq data sets from different cells infected with HCMV. Our overarching findings included the following: (i) different cells generate different HCMV-encoded circRNAs although they also share many common circRNAs (Fig. 1C); (ii) circRNA-encoding sequences are unevenly distributed throughout the HCMV genome; (iii) HCMV circRNAs have consensus sequences flanking BSJ breakpoints, which are similar to the consensus motifs observed in DNA genome-encoded circRNAs from KSHV, EBV, and humans; and (iv) HCMV circRNAs have the canonical “AGN|UNN” signal at the acceptor site and a noncanonical “NAG|GUN” sequence at the donor site (Fig. 2A).

The computationally identified circRNAs were then demonstrated by experiments. First, we employed RT-PCR using specifically designed divergent primers to target the BSJs, as mentioned above. The PCR products were then gel-purified and cloned for DNA sequencing (Fig. 4). The DNA sequences met the circRNA standard. Then, we confirmed the presence of circRNAs using RNase R treatment (Fig. 4H and I). In particular, we identified a circRNA that could be enriched by RNase R treatment (circRNAnumber 5. Fig. 4H and I). We then used AmpFISH to examine the *in situ* circRNA in the HCMV-infected cells. As shown in Fig. 5, because the probes were specific to circRNA and not linear RNAs, the circRNAs were only detected in cells and not degraded by RNase R.

It was reported that circRNAs can regulate the expression of their parental genes. We performed a systematic functional annotation of HCMV circRNAs to determine whether they regulate the expression and viral growth of their parental genes. Previous studies have shown that circRNAs are mainly involved in two functions: as sponges of miRNA in the ceRNA regulatory network and the regulation of parental gene expression (52, 53). For ceRNA network annotation, “DNA replication,” “replication fork,” and 44 additional significant GO/KEGG terms were enriched from the competitive upregulated genes of HCMV circRNAs (Fig. 8). Interestingly, 44 circRNAs were mapped to UL105. It was reported that UL105 interacts with Snapin, leading to modulation of viral DNA synthesis and progeny production (54). Our results highlight the idea that HCMV may hijack host cell DNA synthesis by encoding circRNAs involved in a circRNA-miRNA-mRNA network.

In summary, our current studies not only computationally identified HCMV-encoded circRNAs but also confirmed the existence of HCMV circRNAs in infected cells. We initiated this study not only to explore more components of the HCMV transcriptome but also to elucidate the biological functions of noncoding RNAs in HCMV. Therefore, this is just the beginning of our studies, and future studies will be focused on the following aspects: (i) the role played by circRNAs in latent HCMV infection; (ii) the biological functions of HCMV circRNAs, not only in terms of viral replication and reactivation but also in host cells; and (iii) mutagenesis studies on HCMV circRNA biogenesis.

## MATERIALS AND METHODS

### Data collection.

The RNA sequencing data sets were collected from the NCBI Gene Expression Omnibus (GEO). The total RNA samples were, respectively, prepared from (i) HCMV TB40/E strain infected primary fibroblasts (HFF), endothelial cells (EC), and neural progenitors (NPCs) derived from embryonic stem cells at 48 hpi and 96 hpi (GSE73853) (22); (ii) HCMV HAN-strain-infected human embryonic lung fibroblasts (HELF) cells at 72 hpi with RNase R treatment (GSE138836) (47); (iii) KSHV BCBL1 strain infected B cell lymphoma cells with RNase R treatment (GSE117798) (30); and (iv) EBV Akata strain infected B cell lymphoma cells with RNase R treatment (GSE116675) (13) (Table 1). Lastly, the mRNA and small-RNA were harvested from HCMV Towne strain-infected HFF cells at 72 hpi (GSE63797) (8).

### Splice junction analysis.

Similar to our previously described method (49, 55), the analysis was performed on two Intel W-3175× CPUs with 128 GB memory running on the Ubuntu system (version 18.04). The trimmed reads were aligned with the reference genomes of the TB40/E strain and HAN strain (24) using Python2 script ViReMa (–MicroInDel_Length 5 –Defuzz 0 -FuzzEntry).

### *De novo* circRNA identification and reconstruction.

Raw reads were aligned with the Burrows Wheeler Aligner (BWA) (56) (BWA-MEM version 0.7.17-R1188, Illumina) to the human, HCMV, KSHV, and EBV reference genomes: hg19, TB40 strain (KF297339.1), HAN strain (KJ426589.1), KSHV BCBL1 strain (HQ404500), and EBV Akata strain (KC207813.1). Alignment statistics were performed with Qualimap2 (version 2.2.1) (57). CIRI2 (version v2.0.6) (27) was used for circRNA calling. The reconstruction of partial and full-length circRNAs was performed with CIRI-full (version 2.0) (28).

### Analysis of junction sequence motifs of circRNAs.

Junction sequences (14 nt around the circRNA start and circRNA end) were obtained from the reference genome using bedtools (58). The WebLogo 3 webtool (http://weblogo.threeplusone.com/create.cgi) was used to generate sequence motifs (59).

### Sequence homology analysis.

For each experimentally confirmed BSJ, 60 bp sections around the 5′ and 3′ breakpoints were compared using blastn (BLAST) to identify homologous as well as reverse complementary sequences of 6 bp or longer.

### Functional analysis of the HCMV circRNAs in the competitive endogenous RNA coregulatory network.

Mature human miRNAs were downloaded from the miRbase database (http://www.mirbase.org/), and interactions between HCMV circRNAs and human miRNAs were predicted by miRanda (-sc 150 -en −7) (60). The miRNA target genes were predicted using miRWalk (http://mirwalk.umm.uni-heidelberg.de/). The differential expression (DE) of mRNA or miRNA between each group was selected as 4-fold changes with a *P* value followed by a false discovery rate (FDR) correction of 0.01 using DESeq2 (61). ClusterProfiler (62), which uses a modified Fisher's exact test followed by the Benjamini–Hochberg multiple hypothesis testing correction methods and a corrected *P* value cut off 0.05 was used to perform gene functional annotation clustering by using humans as the background, default option, and annotation categories. Significantly enriched KEGG pathways were identified using a hypergeometric test and Benjamini-Hochberg FDR correction (63, 64).

### Quantification and plotting.

Quantifications and plots were produced using python (version 3.9.0) with the plotly module (https://plotly.com/python/) and the R statistical environment (version 3.4.5) with the R packages gggenes (https://wilkox.org/gggenes/) and ggplot2 (65).

### Cell culture, virus, and infection.

HFF cells (ATCC no. SCRC-1041) were either infected or mock-infected with HCMV Towne (66) expressing a GFP marker at a multiplicity of infection (MOI) of 3 and were cultured in DMEM (GIBCO) supplemented with 5% fetal bovine serum (ExCell Bio, Shanghai, China), penicillin (100 IU/mL)-streptomycin (100 μg/mL), and amphotericin B (2.5 μg/mL, Sigma) at 37°C with 5% CO2. The virus was passaged once in HFF cells to make the virus stock. Total RNA was harvested from cells using TRIzol reagent (Invitrogen).

### Reverse transcription, inverse PCR, and qPCR.

About 4 μg of total RNA or an equal amount of RNA treated with RNase R (Lecigen) was reverse-transcribed with Prime Script TM RT Master Mix (TaKaRa) using random hexamers. The divergent and convergent primers used in this study are summarized in Table 2. Inverse PCR was performed using EasyTaq PCR SuperMix (TransGen Biotech) with 1 μL of diluted cDNA templated at a concentration of 1:20. qPCR was performed with Tip Green qPCR SuperMix in accordance with the manufacturer’s instructions (TransGen Biotech).

### Cloning and identification of circRNAs.

Inverse RT-PCR products were separated on 2% agarose gel. DNA fragments with the candidate BSJ sequences were gel-purified (OMEGA Gel Extraction kit) and TA-cloned (pMD18-T Vector Cloning kit, TaKaRa). At least 8 colonies were checked for the insertion of candidate PCR products by PCR with M13 universal primers and following PCR purification (DNA Clean & Concentrator kit, Zymo), DNA with candidate BSJ sequences was Sanger-sequenced (Sangon Biotech) using M13 universal primers. Sequencing results were then blasted against the HCMV reference genome (Towne strain and HAN strain). The 5′ and 3′ breakpoints of BSJs and FSJs were manually curated so that ambiguous nucleotides could be counted as the donor sequence if they existed around the junction. CircRNAs with BSJ breakpoints that differed within 20 nt were considered variants of one circRNA.

### Amplified fluorescence *in situ* hybridization (AmpFISH).

AmpFISH probes to detect linear or circRNAs of HCMV (Towne strain) or HCR hairpins were designed, labeled, and purified as described previously (32). The probe sequences are shown in Table 2. AmpFISH was also performed as described previously (32). Briefly, MRC5 cells were seeded on the glass coverslips (0.1 mm thickness), coated with 0.1% gelatin, and cultured in DMEM media with 10% fetal bovine serum (FBS) and 1% P/S. Cells were infected with Towne at an MOI of 0.1 and fixed at 48 h after infection with 4% paraformaldehyde/1XPBS for 10 min at RT. The cells were then washed with 1× PBS followed by incubation with 70% ethanol for 10 min at RT. The ampFISH procedure was performed as described previously (32). For RNase R treatment, the cells were fixed with 4% PFA and permeabilized with 70% ethanol and 0.5% Triton X-100 for 10 min at RT. After permeabilization, cells were then equilibrated with 1× reaction buffer (RNase R buffer) for 30 min, followed by incubation with 20 units of RNase R (Lucigen, RNR07250) for 2 to 3 h at 37°C, and then, the ampFISH procedure was applied. About 16 optical sections separated by 0.2 μm with100 to 2,000 ms of exposure time were acquired for each image using a 63× oil immersion objective in an Axiovert 200 M inverted fluorescence microscope (Zeiss, Oberkochen, Germany). The linear and circular RNA molecules in the infected cells were quantified by counting each RNA spot through an image processing program built-in MATLAB (MathWorks, Natick, MA), as described earlier (32).

### Data availability.

The code used in this study and extended data are available from the GitHub repository, https://github.com/ShaominYang/Circular-RNAs-represent-a-novel-class-of-HCMV-transcripts.
