# Supplementary material for: Circular RNAs Represent a Novel Class of Human Cytomegalovirus Transcripts
Source: Microbiol Spectr. 2022 May 23;10(3):e01106-22. doi: 10.1128/spectrum.01106-22 (PMC9241847; doi:10.1128/spectrum.01106-22)
Supplement: SUPPLEMENTAL FILE 1 — Supplemental material. Download spectrum.01106-22-s001.pdf, PDF file, 4.3 MB [file spectrum.01106-22-s001.pdf]

Supplemental Material Information

Authors: Shaomin Yang[1†], Xiaolian Liu[1†], Mei Wang[1†], Di Cao[1†], Dabbu Kumar Jaijyan[2], Nicole Enescu[2], Jian Liu[3], Songbin Wu[4], Sashuang Wang[4], Wuping Sun[4], Lizu Xiao[4], Yaolan Li[1], Hong Zhou[5], Sanjay Tyagi[6,7], Jianguo Wu[1], Qiyi Tang[5], Hua Zhu[2*]

Affiliations:

[1] Jinan University, Guangzhou, Guangdong, 510632, China.

[2] Department of Microbiology and Molecular Genetics, New Jersey Medical School, Rutgers University, 225 Warren Street, Newark, NJ 070101, USA.

[3] School of Biological Sciences and Biotechnology, Minnan Normal University, Zhangzhou 363000, China.

[4] Department of Pain Medicine and Shenzhen Municipal Key Laboratory for Pain Medicine, Shenzhen Nanshan People's Hospital, The 6th Affiliated Hospital of Shenzhen University Health Science Center, Shenzhen, China.

[5] Department of Microbiology, Howard University College of Medicine, 520 W Street NW Washington, DC 20059, USA.

[6]Public Health Research Institute, New Jersey Medical School, Newark NJ07103

[7]Department of Medicine, New Jersey Medical School, Rutgers University.

†Co-first authors, these authors contributed equally to this work.

*Correspondence to: hua.zhu@rutgers.edu

Figure S1. Mapping statistics.

(A-F) Read counts from HCMV TB40/E strain-infected EC **(A)**, NPCs **(B)**, HFF **(C)**, HAN strain infected-HELF **(D)**, KSHV BCBL1 strain-infected-B cell lymphoma **(E),** and EBV Akata strain-infected B cell lymphoma **(F)** related RNA-Seq. **(G-I)** Genome coverage of HCMV HAN strain-infected HELF **(G)**, KSHV BCBL1 strain-infected B cell lymphoma **(H),** and EBV Akata strain-infected B cell lymphoma **(I)**. **(J-K)** Frequency of circularization events in KSHV **(J)** and EBV **(K)**. Counts of BSJ-spanning reads of circRNAs (starting from a coordinate in the x-axis and ending at a coordinate in the y-axis) indicated by color. The circRNA start/stop positions are shown as histograms on the x- and y-axes. Ranked expression levels of *de novo* identified circRNAs from HCMV and human genomes. The labels of the top 10 circRNAs are shown according to their genome positions. The complete list of splice sites is provided in Data S1.

Figure S2. Genome organization and Reconstructed circRNAs from the HCMV HAN strain.

The ORFs are color-coded according to the growth properties of their corresponding virus gene-deletion mutants. Variation multigene segment of the HAN strain: ~15 kb (176,729 to 191,263 bp). The relative expression levels of circRNAs are indicated by the colors at the bottom. Reconstruction of full-length circRNAs and partially assembled circRNAs indicated by “Full” and “Break”, respectively. The complete list of splice sites is provided in Data S1.

Figure S3. Inverse RT-PCR used for subcloning and sequencing.

(A) Inverse RT-PCR result of the HCMV clinical strains TB40/E and Toledo and a laboratory strain with selected primer sets**. (B-C)** The expression of HCMV circRNAs in HFF, HELF**,** and U373 cells at different infection time points. **(D)** Inverse RT-PCR on RNA treated with or without RNase R. GAPDH and HCMV linear RNA, IE1/2, early gene (UL105), and late (UL111A) gene**. (E)** Relative resistance ability to RNase R of linear RNA and circRNAs. The value was normalized to the corresponding sample without RNase R treatment. **(F-I)** BSJ breakpoints are indicated by dashed lines. Donor (green), acceptor (blue) sequences, and

downstream/upstream sequences (grey) flanking the junction were aligned with the BSJ sequence. **(J)** Sanger sequencing result of three different divergent primers amplified BSJ#5 for constructing full-length of circRNA#5.

Figure S4. Demonstration of the high level of specificity of the spot counting algorithm and the detection of human circHIPK3 using smFISH/ampFISH.

(A) Demonstration of high specificity of spot counting algorithm. Analysis of same field of cells as in Figure 5 is presented. A yellow circle is placed over each detected spot. The circles and cell boundaries are placed over a compressed z-stacks of Cy5 images filtered by a Laplacian filter. The right panels represent enlargements of indicated regions from the middle panels that were designed to show the detected spots within each yellow circle. The contrast levels of the underlaying images may appear different from the raw images in Figure 5 because of processing by the Laplacian filter. **(B)** The detection of human circHIPK3 using ampFISH. Linear HIPK3 transcript was detected using a smFISH probe set comprising of 48 probes and circHIPK3 was detected using a ampFISH probe pair against the sequences on either side of BSJ. Images on the left are for the GFP (depicting HCMV) and DAPI channels and ones on the right are merged z-stacks for Cy5 channels for the same fields. The bottom panel shows a pair of cells in which smFISH signal from linear HIPK3 mRNA is shown in green, ampFISH signal from circHIPK3 RNA is shown in red and the nuclei in blue. smFISH probes were designed so that they would not bind in the region of linear HIPK3 RNA that is common between the linear and circular RNA species. In general, no colocalization between the spots of two colors was observed. **(C-D)** Number of mRNA spots corresponding to single mRNA molecules are shown for indicated categories of cells. Two-tailed unpaired t test. *$p < 0.05$, **$p < 0.01$, ***$p < 0.001$ mock vs. infected group; ### $p < 0.001$ without RNase R treatment vs. with RNase R treatment group. Data presented as mean ± S.E.M. The number of cells analyzed (n) are indicated in labels of the bars.

Figure S5. The circRNA-miRNA-mRNA network of HCMV circRNA is involved in the competitive endogenous RNA coregulatory network.

(A) “DNA replication” GO Biological Process term, **(B)** “ATPase activity” GO Cellular Component term, **(C)** “Replication fork” GO Molecular Function term, **(D)** “Cell cycle” KEGG pathway term.

Data S1. BSJs called by CIRI2 and reconstructed circRNAs predicted by CIRI-full

Data S2. Experimentally confirmed HCMV circRNAs, Homology and complementarity analysis of simulated circRNAs and experimentally confirmed HCMV circRNAs

Data S3. Functional annotation of HCMV circRNAs in regulating parental gene

Data S4. Human miRNAs interacting with full-length HCMV circRNAs predicted by miRanda

Figure S1

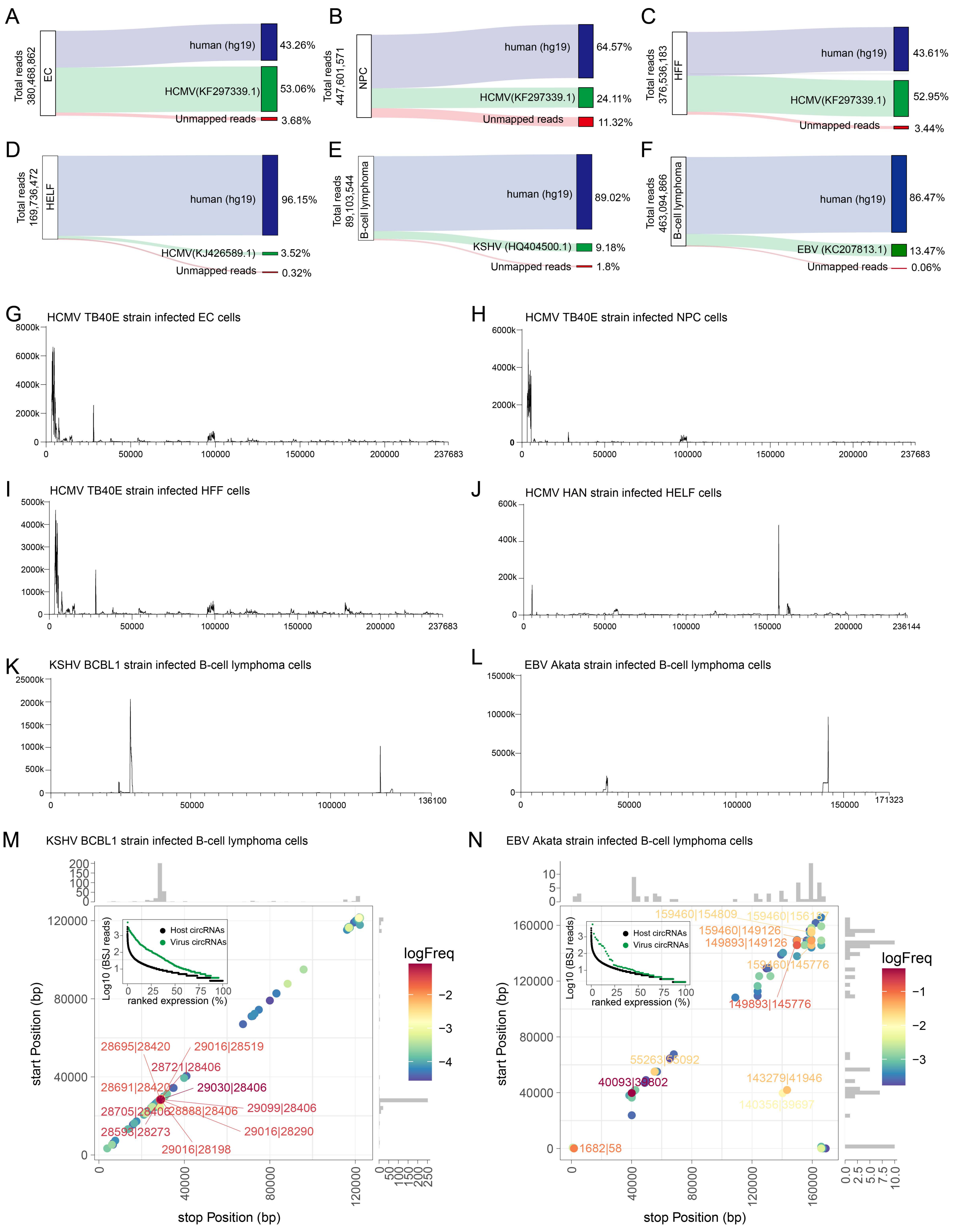

A
Total reads 380,468,862
EC
human (hg19) 43.26%
HCMV(KF297339.1) 53.06%
Unmapped reads 3.68%
B
Total reads 447,601,571
NPC
human (hg19) 64.57%
HCMV(KF297339.1) 24.11%
Unmapped reads 11.32%
C
Total reads 376,536,183
HFF
human (hg19) 43.61%
HCMV(KF297339.1) 52.95%
Unmapped reads 3.44%
D
Total reads 169,736,472
HELF
human (hg19) 96.15%
HCMV(KJ426589.1) 3.52%
Unmapped reads 0.32%
E
Total reads 89,103,544
B-cell lymphoma
human (hg19) 89.02%
KSHV (HQ404500.1) 9.18%
Unmapped reads 1.8%
F
Total reads 463,094,866
B-cell lymphoma
human (hg19) 86.47%
EBV (KC207813.1) 13.47%
Unmapped reads 0.06%
G
HCMV TB40E strain infected EC cells
H
HCMV TB40E strain infected NPC cells
I
HCMV TB40E strain infected HFF cells
J
HCMV HAN strain infected HELF cells
K
KSHV BCBL1 strain infected B-cell lymphoma cells
L
EBV Akata strain infected B-cell lymphoma cells
M
KSHV BCBL1 strain infected B-cell lymphoma cells
start Position (bp)
stop Position (bp)
Log10 (BSJ reads)
ranked expression (%)
Host circRNAs
Virus circRNAs
logFreq
28695|28420
29016|28519
28721|28406
28691|28420
29030|28406
28705|28406
28888|28406
29099|28406
28593|28273
29016|28290
29016|28198
N
EBV Akata strain infected B-cell lymphoma cells
start Position (bp)
stop Position (bp)
159460|154809
159460|156187
159460|149126
149893|149126
159460|145776
149893|145776
55263|55092
40093|39802
143279|41946
140356|39697
1682|58

Figure S2

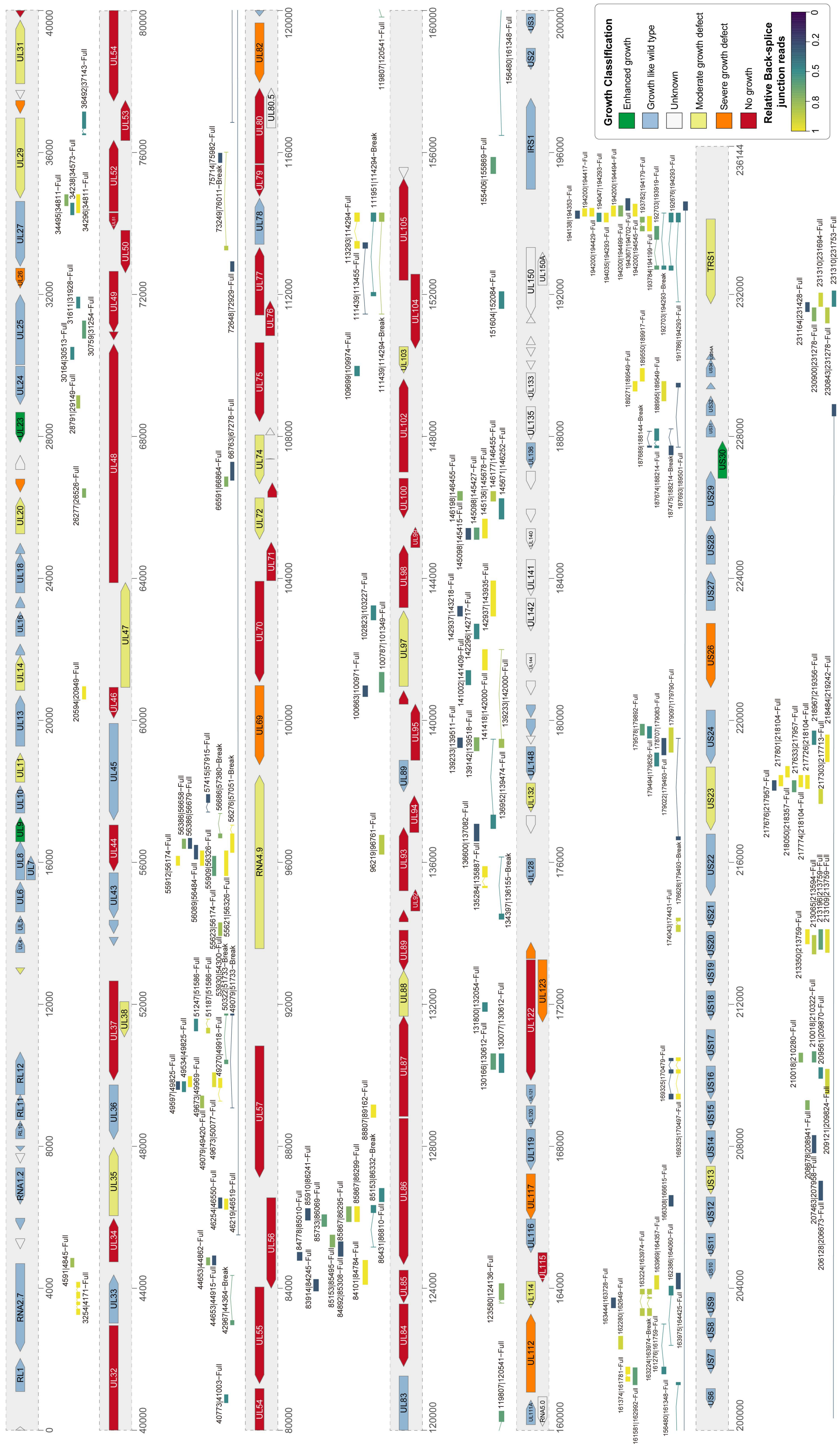
Growth Classification
Enhanced growth
Growth like wild type
Unknown
Moderate growth defect
Severe growth defect
No growth
Relative Back-splice junction reads

Figure S3

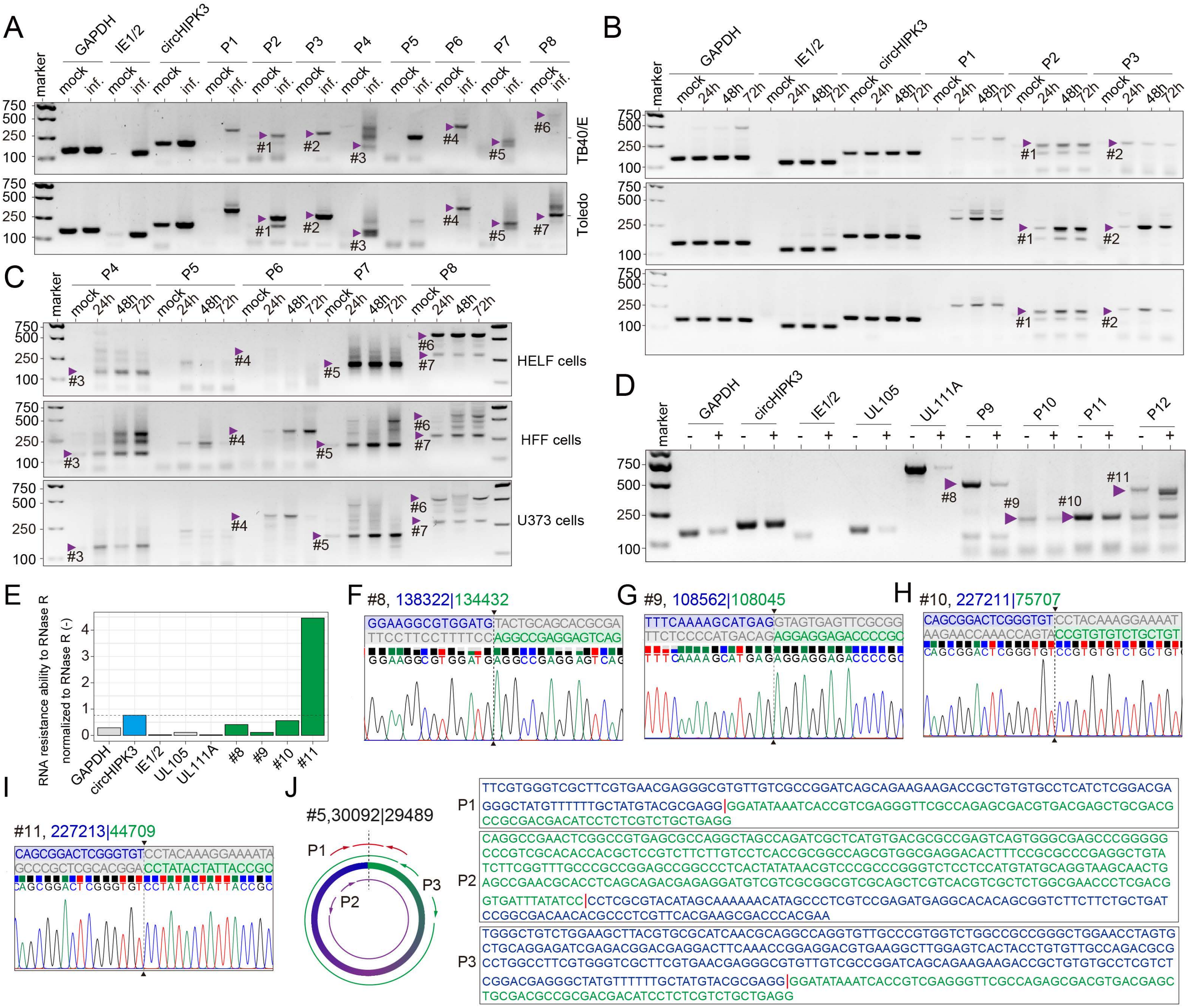
A
marker
GAPDH
IE1/2
circHIPK3
P1
P2
P3
P4
P5
P6
P7
P8
mock
inf.
750
500
250
100
#1
#2
#3
#4
#5
#6
#7
TB40/E
Toledo
B
GAPDH
IE1/2
circHIPK3
P1
P2
P3
mock
24h
48h
72h
HELF cells
HFF cells
U373 cells
C
P4
P5
P6
P7
P8
HELF cells
HFF cells
U373 cells
D
GAPDH
circHIPK3
IE1/2
UL105
UL111A
P9
P10
P11
P12
-
+
#8
#9
#10
#11
E
RNA resistance ability to RNase R
normalized to RNase R (-)
GAPDH
circHIPK3
IE1/2
UL105
UL111A
#8
#9
#10
#11
F
#8, 138322|134432
GGAAGGCGTGGATG
TACTGCAGCACGCGA
TTCCTTCCTTTTCC
AGGCCGAGGAGTCAG
G
#9, 108562|108045
TTTCAAAAGCATGAG
GTAGTGAGTTCGCGG
TTCTCCCCATGACAG
AGGAGGAGACCCCGC
H
#10, 227211|75707
CAGCGGACTCGGGTGT
CCTACAAAGGAAAAT
AAGAACCAAACCAGTA
CCGTGTGTCTGCTGT
I
#11, 227213|44709
CAGCGGACTCGGGTGT
CCTACAAAGGAAAATA
GCCCGCTCGCACGGA
CCTATACTATTACCGC
J
#5,30092|29489
P1
P2
P3
P1
TTCGTGGGTCGCTTCGTGAACGAGGGCGTGTTGTCGCCGGATCAGCAGAAGAAGACCGCTGTGTGCCTCATCTCGGACGA
GGGCTATGTTTTTTGCTATGTACGCGAGG|GGATATAAATCACCGTCGAGGGTTCGCCAGAGCGACGTGACGAGCTGCGACG
CCGCGACGACATCCTCTCGTCTGCTGAGG
P2
CAGGCCGAACTCGGCCGTGAGCGCCAGGCTAGCCAGATCGCTCATGTGACGCGCCGAGTCAGTGGGCGAGCCCGGGGG
CCCGTCGCACACCACGCTCCGTCTTCTTGTCCTCACCGCGGCCAGCGTGGCGAGGACACTTTCCGCGCCCGAGGCTGTA
TCTTCGGTTTGCCCGCCGGAGCCGGCCCTCACTATATAACGTCCCGCCCGGGTCTCCTCCATGTATGCAGGTAAGCAACTG
AGCCGAACGCACCTCAGCAGACGAGAGGATGTCGTCGCGGCGTCGCAGCTCGTCACGTCGCTCTGGCGAACCCTCGACG
GTGATTTATATCC|CCTCGCGTACATAGCAAAAAACATAGCCCTCGTCCGAGATGAGGCACACAGCGGTCTTCTTCTGCTGAT
CCGGCGACAACACGCCCTCGTTCACGAAGCGACCCACGAA
P3
TGGGCTGTCTGGAAGCTTACGTGCGCATCAACGCAGGCCAGGTGTTGCCCGTGGTCTGGCCGCCGGGCTGGAACCTAGTG
CTGCAGGAGATCGAGACGGACGAGGACTTCAAACCGGAGGACGTGAAGGCTTGGAGTCACTACCTGTGTTGCCAGACGCG
CCTGGCCTTCGTGGGTCGCTTCGTGAACGAGGGCGTGTTGTCGCCGGATCAGCAGAAGAAGACCGCTGTGTGCCTCGTCT
CGGACGAGGGCTATGTTTTTTGCTATGTACGCGAGG|GGATATAAATCACCGTCGAGGGTTCGCCAGAGCGACGTGACGAGC
TGCGACGCCGCGACGACATCCTCTCGTCTGCTGAGG

Figure S4

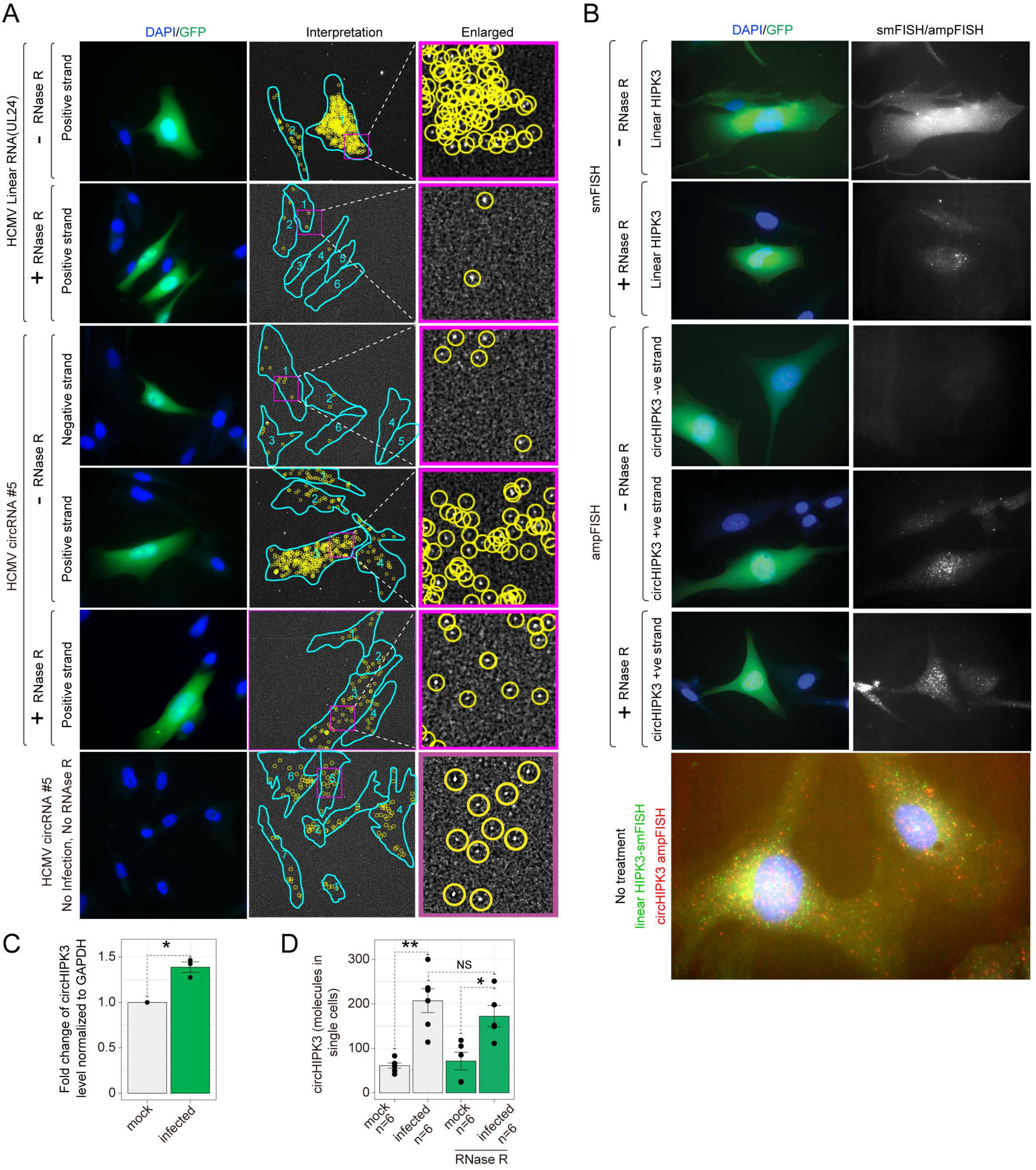
A
DAPI/GFP
Interpretation
Enlarged
HCMV Linear RNA(UL24)
- RNase R
+ RNase R
Positive strand
HCMV circRNA #5
Negative strand
HCMV circRNA #5 No Infection, No RNAse R
B
DAPI/GFP
smFISH/ampFISH
smFISH
ampFISH
Linear HIPK3
circHIPK3 -ve strand
circHIPK3 +ve strand
No treatment
linear HIPK3-smFISH
circHIPK3 ampFISH
C
Fold change of circHIPK3 level normalized to GAPDH
1.5
1.0
0.5
0
mock
infected
*
D
circHIPK3 (molecules in single cells)
300
200
100
0
**
NS
*
mock n=6
infected n=6
mock n=6
infected n=6
RNase R

Figure S5

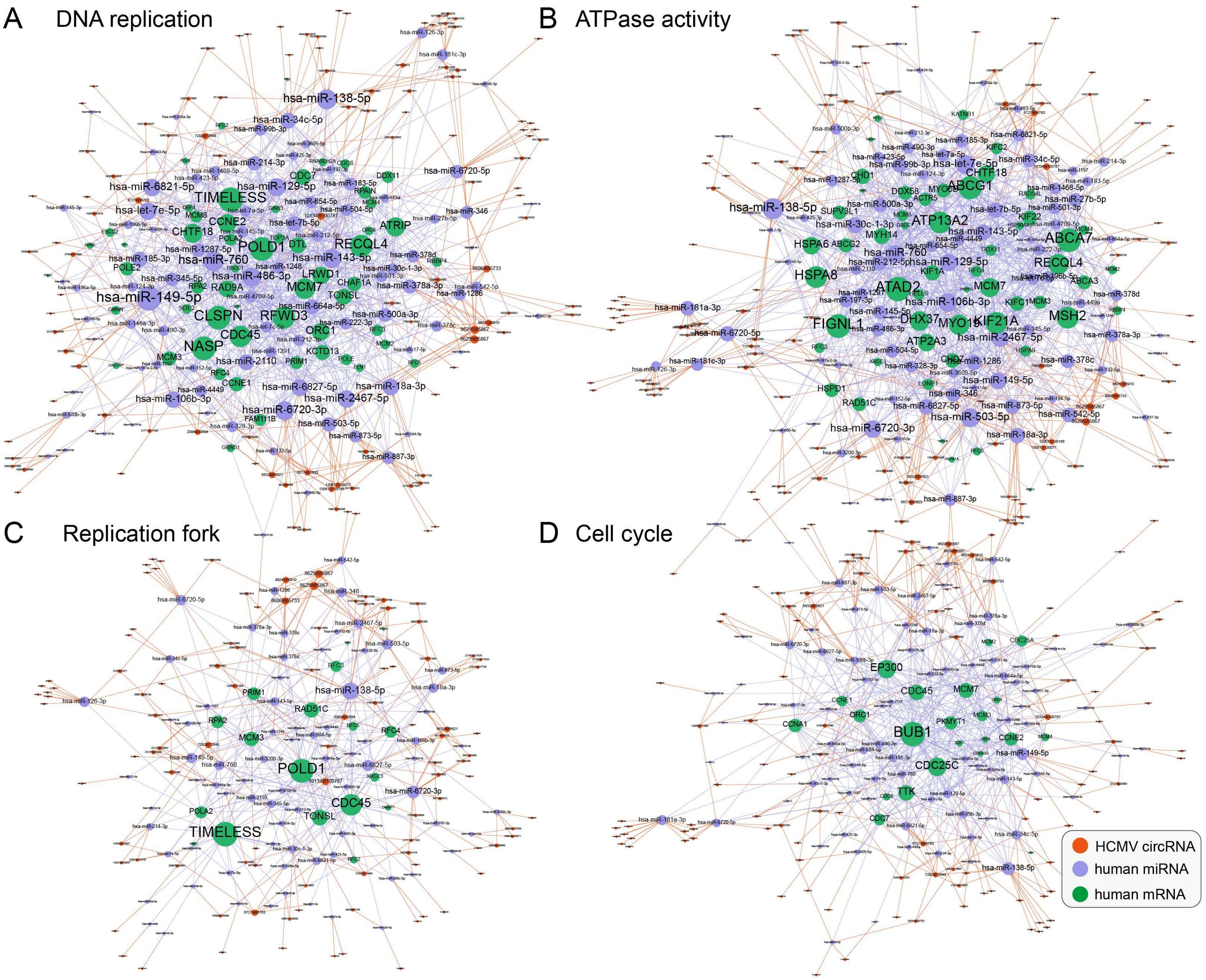
A
DNA replication
B
ATPase activity
C
Replication fork
D
Cell cycle
HCMV circRNA
human miRNA
human mRNA
